# Comparison of Valproic acid Clearance between Epileptic Patients and Patients with Acute Mania

**Published:** 2011

**Authors:** Amir Hooshang Mohammadpour, Mohsen Foroughipour, Mahmoud Reza Azarpazhooh, Mohammad Hasanzadeh Khayat, Saeed Rezaee, Tamara Aghebati, Jamal Shamsara

**Affiliations:** 1 *Pharmaceutical Research Centre and School of Pharmacy, Department of Pharmacodinamy and Toxicology, Mashhad University of Medical Sciences (MUMS), Mashhad, Iran*; 2 *Department of Neurology, Faculty of Medicine, Ghaem Hospital, MUMS, Mashhad, Iran*; 3 *Department of Medicinal Chemistry, Pharmaceutical Research Centre and School of Pharmacy, MUMS, Mashhad, Iran*; 4 *Department of Pharmaceutics, School of Pharmacy, Ahwaz University of Medical Sciences, Ahwaz, Iran*; 5 *Department of Biotechnology, School of Pharmacy, MUMS, Mashhad, Iran*

**Keywords:** Acute mania, Clearance, Epilepsy, Pharmacokinetics, Valproic acid

## Abstract

**Objective(s):**

The purpose of this study was assessment of the influence of acute manic phase on the steady state pharmacokinetics of valproic acid (VPA) in bipolar patients in comparison with those of epileptic patients.

**Materials and Methods:**

Ninteen acutely manic and 25 epileptic patients who fulfilled inclusion and exclusion criteria were entered in this prospective study. Blood samples were collected at trough time in steady state and plasma concentrations were determined by fluorescence polarization immunoassay (FPIA). VPA apparent oral clearance (CL/F) values were calculated in each patient and were compared between groups. As VPA clearance is affected by different factors such as age, total body weight, VPA dosage and the use of concurrent medications, all of these confounding factors were made similar in both groups.

**Results:**

Comparison between two groups showed that CL/F values in acutely manic patients were significantly higher than epileptic patients (10.35±5.77 vs. 7.70±2.63 ml/kg/h, *P*= 0.047).

**Conclusion:**

Acutely manic patients require more VPA dosage to achieve serum concentrations in comparison with those found in epileptic patients. It may be suggested that this increased VPA clearance in acute manic phase may be related to abnormalities in membrane transport systems that may affect on cellular uptake of the drug and its volume of distribution. Since our study is a preliminary investigation in this field, further detailed pharmacokinetic study in acute manic patients are warranted to confirm results of this study.

## Introduction

Valporic acid (VPA) is chemically related to free fatty acids. This drug is one of the most widely used anti epileptic drugs (AEDs) in treatment of both generalized and partial seizures in adults and children (1,2). The capability of treating many types of seizure with a single anticonvulsant has resulted in the wide-spread use of VPA particularly in children (3,4). Furthermore it is increasingly used for therapy of bipolar mood disorders, neuropathic pain and for prophylactic treatment of migraine headache (5,6). Clinical effects of VPA bear a relatively close relation to serum drug concentration. Significant inter-individual variability has been reported in VPA pharmacokinetic (7) and optimal use of this drug and its appropriate serum concentration depend on different factors such as age, total body weight, VPA dosage and co-administration of the other drugs which affect pharmacokinetics of the VPA (1, 2,8). Other studies report that pharmacokinetics of lithium (Li) and carbamazepine (CBZ) are affected by acutely manic phase in bipolar patients (9-11). Based on the results of these studies, Li and CBZ clearance increase in acutely manic patients. Because of the abnormalities in the neurotransmitters, neuroendocrine and membrane transport in manic patients, hepatic clearance and volume of distribution of these drugs may be affected in acute manic phase (9, 12,13). Based on these considerations, it is suggested that also pharmacokinetic of other drugs such as VPA may be affected in these patients. Despite the wide use of this medication in manic patients, there is no investigation about the influence of acute manic phase on VPA pharmacokinetics. The present prospective study was performed to assess probable difference of VPA pharmacokinetic in acutely manic patients in comparison with epileptic patients.

## Materials and Methods

***Patients***

This study was approved by the Ethics Commission of MUMS (Mashhad University of Medical Sciences). All Patients signed a consent form prior to entry into the study. This study was carried out prospectively during the course of a therapeutic drug monitoring program in the Psychiatric and Neurologic Clinics of Ebn–sina and Ghaem hospitals of Mashhad University of Medical Sciences in between July 2005 and April 2006. Both epileptic and acutely manic patients entered this study and were compared with each other in two different groups. VPA apparent oral clearance (CL/F) values were calculated and compared between these two groups.

 All of the patients fulfilled the following inclusion criteria: a. Diagnosis for epilepsy was approved by EEG, clinical examination and acute mania was assessed according to DSM-IV criteria (1). b. Receiving a constant dose of VPA for at least 5 days. c. Age and total body weight of 16-45 years and 51-75 kg, respectively.

Exclusion criteria were: a. Patients with abnormal renal function tests (serum creatinine> 1.2 mg/dl in adult males and 1.1 in adult females). b. Patients with abnormal liver function tests (AST and ALT > 2.5 folds of normal values). c. Taking carbamazepine, phenytoine, phenobarbital, felbamate, ethusoximide, acyclovir and rifampin. d. History of cardiovascular disorders, renal and hepatic disorders, thyroid disorders, diabetes mellitus, COPD. e. History of bipolar mood disorder in epileptic patients and vice versa. Whenever a blood sample was taken, all relevant demographic data (e.g. age, gender, body weight) and medication details (sampling time, duration of therapy, concurrent medication and adverse drug reactions) were recorded. In addition, several laboratory tests (CBC, BUN, ALT, AST) performed.

***Blood sampling and drug assays***

Through serum samples were taken before the administration of the morning dose. A fluorescence polarization immunoassay (FPIA) method was used for determination of the serum VPA concentration. An acceptable VPA assay calibration curve should meet the following criteria: Polarization Error (PERR) -2.00 to +2.00 for all calibration and root mean squared error (RMSE) less than or equal to 1.00. 

***Pharmacokinetic and statistical analyses***

Apparent CL/F was calculated for each patient by using the following equation:

(CL/F L/kg/ hr)= VPA dose (mg/kg)/ [Css(mg/l)× T)

Where CL is the total body clearance of drug, F is the oral bioavailability and Css reflected trough concentrations, and so calculated CL/F may represent overestimates of the actual values. All data were entered into a database and analyzed by the use of SPSS software for windows (version 11.5, ). For comparison between the two groups, two-Independent sample T test was used. P value less than 0.05 was consider significant.

## Result

***Characteristics of the study populations***

The study population consisted of 25 epileptic and 19 manic patients who were similar in age, body weight, and use of concurrent medications. Demographic and medication details for the patients are summarized in Table 1. 

***Comparison of VPA CL/F values between epileptic and manic patients***

**Figure 1:**
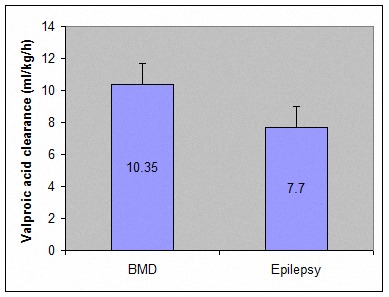
VPA CL/F values in manic and epileptic patients

VPA CL/F between these two groups was compared and VPA clearance was significantly higher in patients with acute mania. Results have been shown in Table 2 and Figure 1.

***Comparison of VPA CL/F values between male and female***

VPA CL/F values between male and female were compared and no significant difference was noted. Results have been shown in Table 3. Also there were no statistically significant correlation between VPA clearance of patients and patients’ age or weight.

**Table 1:** Characteristics of the study population

	Epileptic patients	Manic patients
Patients (n)	25	19
Age (year)	26.36±9.92^1^	38.11±14.82^1^
Total body weight (kg)	62.28±7.38^1^	67.58±7.08^1^
Male/ Female ratio	0.81	1.37
VPA dosage (mg/kg/day)	8.00±1.64^1^	8.81±1.16^1^
Serum VPA^2^ concentration (µg/ml)	42.77±15.93^1^	60.45±29.52^1^

1. Means±SD

2. Valproic acid

**Table 2:** Comparison of VPA CL/F values between two groups

	Epileptic patients	Manic patients	*P* value^3^
VPA CL/F^1^(ml/kg/hr)	7.70±2.63^2^	10.35±5.77	0.047

1.Valproic acid clearance / bioavailability

2. Means±SD

3. Independent sample T test

**Table 3:** Comparison of VPA CL/F values in male and female

	Male group	Female group	*P* value^3^
Patients (n)	20	24	
VPA CL/F^1^(ml/kg/h)	7.92±2.81^2^	9.95±5.69	0.131

1. Valproic acid clearance / bioavalibility

2. Means±SD

3. Independent sample T test

## Discussion

In this study the influence of gender on VPA clearance was evaluated and it was found that there was no significant difference in VPA clearance between males and females. Other studies supported this result (14). By using above approach the confounding effects of these factors were reduced and the difference in VPA CL/F values between these two groups would be affected by the type of the disorder. Because of lacking TDM in and proper response of patients in Iranian hospitals with relatively low dose of VPA, the VPA was administered in doses lower than recommended doses.

The results of this study showed that acutely manic patients had significantly higher VPA clearance compared with epileptic patients. Based on these results manic patients require higher VPA doses to achieve effective serum concentration compared with epileptic patients. As there is no previous study regarding VPA, similar reports of pharmacokinetic changes of the other neuropsychiatric drugs in acutely manic patients will be discussed. Two studies reported that Li and CBZ clearance increased in acutely manic patients (9,15). Two hypotheses for these pharmacokinetic changes would be suggested. 

The first hypothesis is related to hepatic clearance changes due to increase in hepatic blood flow. In this regard, the monoamine hypothesis suggests a functional excess of catecholamine (primary NE and DA) and deregulation between these neurotransmitters which may have an important role in development of acute manic phase in bipolar patients. Beside, disturbance in hypothalamic- pituitary – thyroid axis may be involved in pathophysiology of manic patients. Excess thyroid activity may induce a manic episode by inducing of β-adrenergic activity (13,16). Therefore, acutely manic patients may have an increased catecholamine and sympathetic activity cardiac out put and tissue perfusion. Increased hepatic blood flow may affect hepatic clearance of some drugs. VPA is eliminated almost completely by means of hepatic metabolism, and it has a low hepatic extraction ratio too. In this case the hepatic clearance rate is described with the classic relation used to describe hepatic clearance: Cl_H_= [LBF.(f _B_ Cl _int_ )]/(LBF +f_B_ Cl _int _) where LBF is liver blood flow, f_B _ is the unbound fraction of drug in the blood, and Cl _int_ is the intrinsic ability of the enzyme system to metabolize the drug. Because VPA has a low hepatic extraction ratio, hepatic clearance of this drug may be less affected by hepatic blood flow and this expression for hepatic clearance simplifies to Cl _H _= f _B_ Cl _int_ (1,17). Therefore, it is suggested that increased VPA clearance in manic patients may not be related to changes in hepatic blood flow. 

The second hypothesis related to probable changes in the volume of distribution (V_d_) of drugs in the manic patients. To explain this, it is worth mentioning that there are abnormalities in membrane transports and secondary messenger systems in bipolar patients which results in reduction of erythrocyte Na^+^/ K^+^/ATPase activity (13,16). These studies also suggested that some of the active transporters in cell membranes may be affected in manic patients. Therefore, the cellular uptake of drugs and their volume of distribution may be changed in these patients as well (9, 10,15).

As mentioned above, VPA also has low extraction ratio, so increase in hepatic blood flow in the manic patients has no significant effects on the clearance of this drug. Therefore it is suggested that the change in clearance values in manic patients may be related to change in volume of distribution of this drug. 

## Conclusion

Results of present study showed that acutely manic patients require more VPA dosage to achieve serum concentrations compared with those found in epileptic patients. As in this study only clearance values were compared between these two groups of patients, more detailed investigation (with larger number of patients and through population based analysis) about probable differences of VPA volume of distribution in acute manic phase is suggested. 
